# Systemic Mastocytosis, KIT and the Effects of KIT Tyrosine Kinase Inhibitors on Mast Cell and Basophil Activation

**DOI:** 10.1007/s11882-026-01257-6

**Published:** 2026-02-17

**Authors:** Peter Valent, Karin Hartmann, Gabriele Stefanzl, Yüksel Filik, Karin Bauer, Lina Degenfeld-Schonburg, Karoline V. Gleixner, Frank Siebenhaar, Marek Niedoszytko, Wolfgang R. Sperr, Narges Aghaallaei, Gregor Hoermann, Vito Sabato, Alberto Orfao, Michel Arock, Cem Akin

**Affiliations:** 1 Department of Internal Medicine I, Division of Hematology & Hemostaseology, Medical University of Vienna, Vienna, Austria; 2 Ludwig Boltzmann Institute for Hematology and Oncology, Medical University of Vienna, Waehringer Guertel 18-20, Vienna, A-1090 Austria; 3 Division of Allergy, Department of Dermatology, University Hospital Basel and University of Basel, Basel, Switzerland; 4 Department of Clinical Research, University Hospital Basel and University of Basel, Basel, Switzerland; 5 Department of Biomedicine, University Hospital Basel and University of Basel, Basel, Switzerland; 6 Institute of Allergology, Charité - Universitätsmedizin Berlin, Freie Universität Berlin and Humboldt-Universität zu Berlin, Berlin, Germany; 7 Fraunhofer Institute for Translational Medicine and Pharmacology ITMP, Immunology and Allergology, Berlin, Germany; 8 Department of Allergology, Medical University of Gdansk, Gdańsk, Poland; 9 MLL Munich Leukemia Laboratory, Munich, Germany; 10 Faculty of Medicine and Health Sciences, Department of Immunology, Allergology, Rheumatology and the Infla-Med Centre of Excellence, University of Antwerp, Antwerp, Belgium; 11 Department of Medicine, Servicio Central de Citometria (NUCLEUS), Centro de Investigacion del Cancer (IBMCC, CSIC/USAL, Instituto Biosanitario de Salamanca (IBSAL), University of Salamanca, Salamanca, Spain; 12 Department of Hematological Biology, CEREMAST, Pitié- Salpêtrière Hospital, AP-HP, Paris Sorbonne University, Paris, France; 13 Division of Allergy and Clinical Immunology, University of Michigan, Ann Arbor, MI USA

**Keywords:** Mast cells, Basophils, Mastocytosis, *KIT* mutations, Mast cell, Activation Syndrome, Anaphylaxis, KIT Tyrosine Kinase Inhibitors

## Abstract

**Background:**

Systemic mastocytosis (SM) is a protean hematologic disorder characterized by uncontrolled expansion and accumulation of tissue mast cells (MC) in various organs, including skin, bone marrow, spleen, and gastrointestinal tract. In most cases, neoplastic cells exhibit the transforming *KIT* mutation D816V. Clinical symptoms arise from organ infiltration by neoplastic MC and/or pro-inflammatory mediators and cytokines released by these cells. Indeed, in a majority of the patients, acute or chronic mediator-induced symptoms are recorded, and in some instances, symptoms are severe and drug-resistant or even manifest as life-threatening anaphylaxis.

**Purpose of review:**

In this article, we review the role of MC and basophils in anaphylactic reactions in patients with SM and the impact of genetic variables, co-morbidities, specific IgE, and factors counteracting MC activation.

**Recent findings and summary:**

Whereas avoidance of all known and potential triggers of MC activation is crucial in the management of anaphylaxis in SM patients, specific therapy is also standard, including histamine receptor blockers, other anti-mediator-type drugs and drugs suppressing MC activation and/or expansion. Novel KIT D816V-targeting drugs can potentially decrease the anaphylaxis risk by reducing the MC burden and by targeting KIT-dependent and IgE-receptor-dependent signaling processes in neoplastic MC. Application of such drugs often leads to sustained responses and an increase in the quality of life in these patients.

## Introduction

**Fig. 1:**
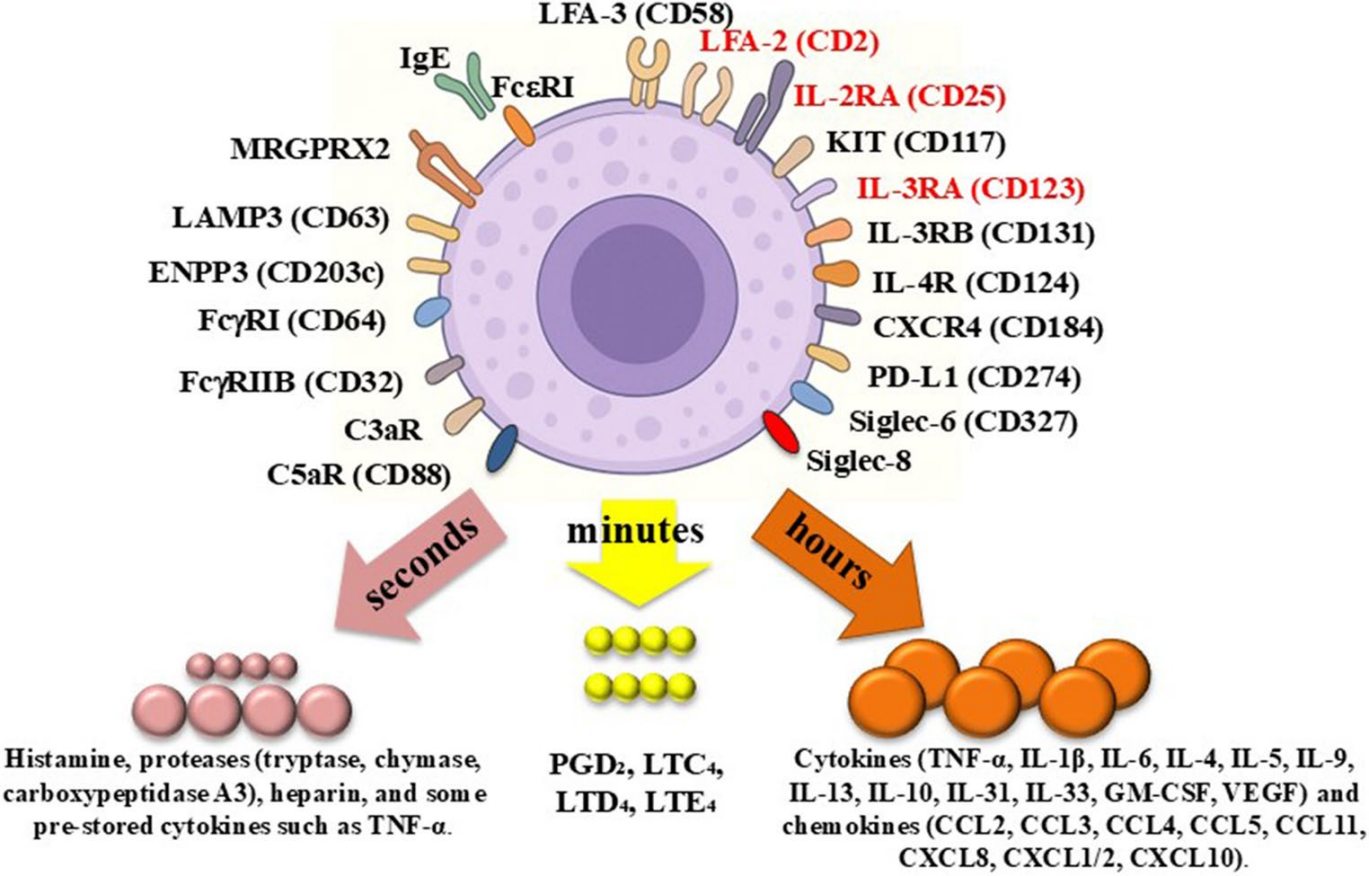
Cell surface antigens expressed on mast cells (MC) in healthy individuals and/or in patients with systemic mastocytosis (SM) and mediators released by these cells upon activation Independent of the organ sites, underlying disease and activation status, MC express the stem cell factor receptor KIT (CD117) which is indispensable for MC differentiation and survival, and is mutated in SM, and the high-affinity IgE receptor (FcεRI) a key receptor mediating allergen-induced MC activation. In addition, MC typically express several cytokine and chemokines receptors, such as the interleukin-4 receptor (IL-4R=CD124) and the chemokine receptor for stromal cell-derived factor-1, CXCR4 (CD184). Neoplastic mast cells in SM display additional surface receptors in an aberrant manner, including the leukocyte function antigen-2 (LFA-2 = CD2), IL-2RA (CD25), and IL-3RA (CD123) (red color). Other cell surface antigens on MC are activation-linked cell surface antigens, including the C3aR and C5aR (CD88), lysosomal membrane antigen 3 (LAMP3 = CD63), and ENPP3 (CD203c). In addition, MC express FcgRI (CD64), FcgRIIB (CD32), Siglec-6 (CD327), and Siglec-8. Upon activation by an allergen (through IgE and FcεRI), mast cells release their preformed mediators within seconds from their cytoplasmic granules. These mediators include histamine and tryptase, and in certain MC subtypes (MC_TC_) also chymase and carboxypeptidase A3. MC also produce, store and release heparin and TNF. Within minutes, activated MC also generate arachidonic acid-derived lipid mediators, such as prostaglandin D₂ (PGD₂), thromboxane A₂, leukotriene C₄ (LTC₄) and its metabolites LTD₄, LTE₄, and LTB₄. Finally, a few hours after activaction, MC synthesize and secrete cytokines and chemokines that exert profound effects on the immune system and tissue homeostasis. These include pro-inflammatory cytokines (TNF, IL-1β, IL-6), Th2-cytokines (IL-4, IL-5, IL-9, IL-13), regulatory cytokines (IL-10), and hematopoietic and angiogenic growth factors (IL-3, GM-CSF, VEGF). MC-derived chemokines include CCL2 (MCP-1), CCL3 (MIP-1α), CCL5 (RANTES), CCL11 (eotaxin-1), CXCL8 (IL-8), and CXCL10 (IP-10). All these molecules are responsible for the immediate and delayed symptoms after MC activation in patients with SM and/or allergies. Abbreviations: CCL, C-C chemokine ligand; CXCL, C-X-C chemokine ligand; ENPP3, ectonucleotide pyrophosphatase/phosphodiesterase 3; GM-CSF, granulocyte-macrophage colony-stimulating factor; IL-, interleukin-; LFA, lymphocyte function–associated antigen; MRGPRX2, Mas-related G protein–coupled receptor member X2; Siglec, sialic acid–binding immunoglobulin-like lectins; TNF, tumor necrosis factor; VEGF, vascular endothelial growth factor

Mast cells (MC) and blood basophils (BA) are critical effector cells in local and systemic hypersensitivity reactions [1–10]. Both cells generate and store numerous vasoactive and inflammatory mediator-molecules, including histamine, arachidonic acid-derived lipid mediators, chemokines and cytokines [1–10]. During an allergic reaction, these mediator-substances are released from MC and/or BA thereby triggering the clinical symptoms of anaphylaxis [3–8]. MC and BA exhibit various cell surface receptors that are involved in cell activation, such as the IgE receptor (FceRI), complement (C3a/C5a) receptors and receptors for certain cytokines or chemokines (Fig. 1; Table 1) [1–15]. Activation of these receptors by endogenous ligands, such as C5a, chemokines, or an allergen through specific IgE and FceRI, usually leads to mediator release in MC and BA [1–11]. Figure 1 provides an overview of cell surface molecules and receptors expressed on MC and BA.

**Table 1:** Cell surface antigens expressed on mast cells (MC) and basophils (BA) in healthy individuals and in patients with systemic mastocytosis (SM)

Antigen/Receptor	Expressed on the surface of					Mediates cell adhesion	Mediates cell activation
(R)	CD	n MC	SM MC	n BA	SM BA		
FceRI	n.c.	+	+ (+/-)*	+	+	-	+
FcgRIIB	CD32B	+/-	-	+/-	+	-	+/-**
Fc(ooo)RI	CD64	-	+	-	-	-	+
C5aR	CD88	+/-	+	+	+	-	+
C3aR	n.c.	+/-	+	+	n.k.	-	+
MRGPRX2	n.c.	+/-	+/-	+	+/-	-	+/-
LFA-2	CD2	-	+ (+/-)*	-	-	+	-
LFA-3	CD58	+	+	+	n.k.	+	-
LAMP3	CD63	+	+	+	+	-	+/- ***
ENPP3	CD203c	+/-	+	+	+	-	+/- ***
IL-2RA	CD25	-	+	+	+	-	-
KIT/SCFR	CD117	+	+	-	-	+/-	+
IL-3RA	CD123	-	+	+	+	+/-	+
IL-3RB	CD131	+/-	+/-	+	+	+/-	+
IL-4R	CD124	+/-	+/-	+	+	-	-
CXCR1	CD128a	-	n.k.	+	n.k.	-	+
CXCR2	CDw128b	-	n.k.	+	n.k.	-	+
CXCR4	CD184	+	+	+	+	+	-﻿
PD-L1	CD274	+	+	+	+	-	-
Siglec-6	CD327	+	+	+	+	-	+**
Siglec-8	n.c.	+	+	+	+	-	+**

*In patients with SM in whom mast cells are very immature, such as in mast cell leukemia, some of the differentiation-related surface antigens, including FceRI and CD2 may not be expressed on mast cells. **Under certain circumstances these cell surface antigens may also mediate or indicate a suppressive effect on cell activation. ***These cell surface antigens increase during cell activation and therefore serve as activation-indicating biomarkers

*n* *MC* normal mast cells, *n* *BA* normal basophils, *n*.*c*. not (yet) clustered, *n*.*k*. not known, *C5aR* complement receptor 5a, *MRGPRX2* Mas-related G protein-coupled receptor X2, *LFA* leukocyte function antigen, *LAMP3* lysosome-associated membrane glycoprotein 3, *ENNP3* ectonucleotide pyrophosphatase/phosphodiesterase 3, *IL*-*2* interleukin-2, *SCFR* stem cell factor receptor, *CXCR *C-X-C chemokine receptor, *PD*-*L1* programmed death ligand 1

Score: +, expressed on all cells; +/- expressed partially (not on all cells), weakly, or only under certain circumstances; -, not detectable on the surface by flow cytometry

MC and BA are hematopoietic cells that are replenished throughout lifetime from immature multipotent hematopoietic stem cells and lineage-restricted progenitors [1, 9, 11, 16–20]. Whereas MC are tissue-resident cells in various organ systems, BA are usually circulating in the peripheral blood [1–7]. Both, MC and BA as well as their precursor cells, can also be detected in the bone marrow (BM). However, whereas most BA complete their development in the BM, immature MC progenitors leave the BM, circulate in the peripheral blood stream and, after trans-migration through the endothelial layer of blood vessels, these cells complete their differentiation and maturation in extramedullary organs [1, 4, 6–11].

Growth and survival of MC and their precursor cells are regulated by stem cell factor (SCF), a tissue-produced cytokine, and its receptor KIT (CD117) expressed by stem cells and MC (Table 1) [1, 9, 11, 19–24]. Moreover, SCF promotes activation and mediator release by MC [25–27]. SCF-induced development of MC is further augmented by other cytokines, such as interleukin-4 (IL-4) or IL-6 [1, 4, 6, 9, 28–30]. Interleukin-3 (IL-3) also supports the expansion of human MC-committed stem- and progenitor cells, but has no or only little effects on normal mature human MC [28–31]. However, IL-3 is a major regulator of growth, differentiation and function of human BA [32–37].

The lifetime of MC and BA vary greatly. Whereas mature MC can survive in culture or local tissue sites for weeks, months, or even years, BA usually disappear several days after having entered blood and subsequently peripheral organs [1, 7, 9, 37–40].

Normal and neoplastic MC produce and store a variety of clinically relevant vasoactive and pro-inflammatory mediators which are released during IgE-dependent or IgE-independent anaphylactic reactions [1–6, 8–11]. These mediators include histamine, prostaglandin D2, leukotrienes, vascular endothelial growth factor (VEGF), chemokines and cytokines such as tumor necrosis factor (TNF) (Fig. 1). Other mediators, such as tryptase, chymase, or heparin, are also released from activated MC (Fig. 1). Some of these mediators, like tryptase or histamine and their metabolites, also serve as diagnostic markers of MC activation [1–6, 8–10].

### Diagnosis, Classification and Management of Systemic Mastocytosis (SM)

SM is a group of hematologic neoplasms characterized by an abnormal expansion and accumulation of neoplastic MC in one or more organ systems, including the BM, lymph nodes, spleen, skin, and the gastrointestinal (GI) tract [5, 8, 41–50]. The BM is almost always involved whereas other organs are variably affected. Most SM patients are adults whereas in children SM is rarely diagnosed. Like in other myeloid neoplasms, all clonal cells in SM, including MC, are derived from neoplastic stem cells [51]. These immature CD34+/CD38- cells can replenish all neoplastic MC and maintain the disease for unlimited time via their self-renewal capacity, whereas clonal MC themselves do not have self-renewal and long-term proliferative capacity but die after some time [51].

Clinical problems in SM may arise from massive organ infiltration by MC, typically seen in advanced SM, and/or from mediator-induced symptoms recorded across all SM variants [5, 8, 42–50]. In many patients, symptoms are chronic and manageable, whereas in others, the symptoms are episodic and severe or even life-threatening [43, 52–54].

In general, SM can be divided into indolent (non-advanced) variants and advanced forms of SM according to the classification of the World Health Organization (WHO) (Table 2) [41–43, 45, 47]. Indolent SM (ISM) is defined by `hematologic stability´. In fact, patients with ISM have a good prognosis and an almost normal life expectancy [41–47]. In most patients with smoldering SM (SSM), the prognosis is also favorable, although some patients may progress during follow up. BM mastocytosis (BMM) is diagnosed in patients with non-advanced disease without skin lesions and a generally low disease burden. Although anaphylactic events are particularly detected in this variant of SM, the overall prognosis is favorable. By contrast, patients with advanced SM, including SM with an associated hematologic neoplasm (SM-AHN), aggressive SM (ASM), and MC leukemia (MCL), have a poor prognosis with reduced survival (Table 2) [41–50]. These patients are candidates for treatment with KIT-targeting drugs or other anti-neoplastic therapies.

**Table 2:** WHO classification of mastocytosis and indications for KIT TKI therapy

Variant and subvariant	Estimated prevalence of KIT D816V*	Indications for KIT D816V TKI therapy (treatment line)
Non-Advanced forms of SM		
Bone marrow mastocytosis (BMM)	90%	drug-resistant anaphylaxis** (second or third line)
Indolent SM (ISM)	90%	drug-resistant anaphylaxis** severe skin lesions**(second or third line)
Smoldering SM (SSM)	95%	drug-resistant anaphylaxis** severe skin lesions**(second or third line)
Advanced forms of SM		
SM with an associated hematologic neoplasm (AHN)	95%	organ damage by SM and/or KIT D816V+ AHN cells*** (first line)
Aggressive SM (ASM)	80-90%	organ damage by SM*** (first line)
Mast cell leukemia (MCL)	50-70%	organ damage by SM***(first line)
Special sub-variants of SM		
Well-differentiated SM	10%	organ damage by SM**** or mediator-symptoms not optimally controlled by antimediator type drugs**
Advanced SM with wild type KIT	0%	organ damage by SM****
Familial SM	10%	organ damage by SM****
Mast cell sarcoma (MCS)	10%	leukemic spread toMCL***,****

*The estimated prevalence in various subsets is based on the available literature and personal experience of co-authors. **Mast cell mediator-induced symptoms are not optimally controlled by anti-mediator drugs. ***In patients with rapidly progressing (leukemia) SM or SM-AHN, KIT TKI may be applied together with other anti-neoplastic therapy such as poly-chemotherapy or hematopoietic stem cell transplantation. ****In many such patients, KIT D816V is not detectable: in these cases, imatinib or masitinib may also be considered. Abbreviations: *WHO* world health organization, *TKI* tyrosine kinase inhibitor, *SM* systemic mastocytosis

### Role of KIT and *KIT* Mutations in the Pathogenesis of SM

KIT is a class III transmembrane tyrosine kinase receptor that is critically involved in MC development. A detailed description of the structures of wild type *KIT* and mutated *KIT* and of relevant KIT downstream signaling pathways is beyond the scope of this article. We refer the interested reader to the available literature [55–59].

Activating mutations in *KIT* are prominent drivers in mastocytosis and serve as a diagnostic hallmark and criterion of SM [41, 45, 47, 55–59]. These mutations mediate SCF-independent MC differentiation and activate multiple oncogenic signaling machineries in neoplastic cells [55–59]. In children, where the disease is usually classified as cutaneous mastocytosis (CM) while SM is rarely found, several different *KIT* mutations (some of them in germline) can be detected [50, 55–59]. It is worth noting that some of these KIT mutant forms are sensitive against imatinib. By contrast, the most prevalent *KIT* mutation in children and adults, D816V, confers resistance against imatinib [55–60]. In children, *KIT* D816V is frequently detected in the rare SM subtype and in about half of the cases with CM carrying a *KIT* mutation – while the remaining cases harbor other *KIT* mutations, often in exons 8 and 9 encoding the extracellular domain of KIT [55–58]. Whereas SM in children is frequently persistent until adulthood, CM often resolves spontaneously around puberty. In adults, *KIT* D816V is detectable in a vast majority of all patients with SM [55–58]. However, major differences are found when comparing certain disease variants (Table 2). For example, *KIT* D816V is detectable in ≥ 90% of all patients with typical ISM and SSM. By contrast, in patients with MCL, well-differentiated SM and MC sarcoma, the prevalence of *KIT* D816V is lower (Table 2). Overall, the aggressiveness of SM does not correlate with the presence of *KIT* D816V or other *KIT* mutations in adults. Similarly, the course of childhood disease (CM or SM) is not dictated by the type of *KIT* mutation although *KIT* D816V may be more prevalent in SM than in CM [61]. Overall, most childhood patients with CM or SM have stable disease and an excellent prognosis regardless of the presence and type of *KIT* mutation. Nevertheless, rare cases with aggressive SM or even MCL have been described in children. Depending on the type of SM, the mutant form of *KIT* is not only detected in MC and MC-committed progenitor cells but also in other myeloid lineages, including BA [62, 63]. Especially in smoldering SM and advanced SM, BA are part of the malignant clone. This observation is consistent with the concept that SM is a neoplasm of clonal hematopoietic stem cells that are committed to give rise to MC and other myeloid cells in various tissues and organs [9, 48, 51].

### KIT-targeting Tyrosine Kinase Inhibitors (TKI) in SM

Several TKI targeting KIT D816V in neoplastic MC have been developed over the past 20 years [46, 49, 50, 60, 64–72]. Midostaurin is a multi-targeted TKI that blocks KIT D816V and suppresses growth of KIT-transformed MC as well as IgE-dependent mediator release from MC and BA [60, 64–66, 71, 72]. Although midostaurin has shown promising clinical efficacy in a global trial, patients with advanced SM often exhibit or acquire resistance [46, 64]. In recent years, several additional TKI directed against KIT D816V have been developed [46, 67–70, 73, 74]. Avapritinib has been described as a more selective and more potent inhibitor of KIT D816V compared to midostaurin [67–70]. Other novel KIT D816V-targeting drugs, such as bezuclastinib or elenestinib, are currently being tested in clinical trials [73, 74]. Because of their efficacy in interfering with MC expansion and activation, as well as their tolerability, some of these TKI are useful in the treatment of patients with non-advanced, symptomatic SM [75–77]. Other TKI, such as imatinib or masitinib, target wild type KIT and some other mutant forms of KIT, but not KIT D816V [57–60, 78–80]. Thus, these drugs may be useful agents in patients with KIT D816V-negative SM, including cases with well differentiated SM (WDSM) and familial mastocytosis [78–82]. Table 3 shows a summary of KIT-targeting TKI currently used and/or explored in the context of SM.

**Table 3:** KIT tyrosine kinase inhibitors (TKI) and their effects on mast cell activation in patients with systemic mastocytosis

Drug	Major molecular targets	Inhibits mast cell or basophil activation	Mechanism of drug action
Imatinib	KIT WT, KIT K509I	no	-
Masitinib	KIT WT, KIT K509I	n.k.	-
Midostaurin	KIT WT, KIT K509I, KIT D816V, other KIT mutant forms, BTK, SYK, PKC	yes	SYK inhibition PKC inhibition
Avapritinib	KIT WT, KIT K509I, KIT D816V, other KIT mutant forms,	yes	Very strong effect on KIT and KIT mutant forms
Bezuclastinib	KIT WT, KIT K509I, KIT D816V, other KIT mutant forms,	n.k.	-
Elenestinib	KIT WT, KIT D816V	n.k.	-

*KIT WT* wild type KIT, *BTK* Bruton´s tyrosine kinase, *SYK* spleen tyrosine kinase, *PKC* protein kinase C, *n*.*k*., not known

### Mechanisms of MC Activation and BA Activation in SM

Although clonal *KIT*-mutated BA may be detected, their role in mediator-induced symptoms in SM remains uncertain. Rather, most symptoms recorded in such patients may be related to MC activation [5, 8, 43, 50, 52, 53]. Several cell surface antigens, genetic factors, disease-related variables and patient-related factors may contribute to MC- and BA activation in SM [5, 8, 48, 50, 52, 53]. An interesting aspect is that KIT D816V, although acting as a driver of MC differentiation and MC accumulation, is not a major trigger of MC activation which is in line with the observation that many patients with SM are free of symptoms over a long period of time [47–50]. Rather, most clinically relevant events related to severe MC- or BA activation in SM are based on IgE-dependent reactions (induced for example by Hymenoptera venom) or idiopathic allergic reactions [83–85]. It is worth noting in this regard that in patients with SM, including advanced SM, clonal BA and clonal MC usually display functional IgE receptors and other relevant surface receptors such as functional C5a receptors (Table 1) [11, 14, 86, 87]. However, in patients with MCL and a very immature MC morphology, MC may exhibit low levels of or even lack IgE receptors and C5a receptors (Table 1) [88, 89]. In these patients, MC activation-related symptoms may be due to other mechanisms such as constitutive mediator release from a very high MC burden or direct drug-induced toxic cell damage (e.g., by cytostatic drugs) [90, 91]. There are also other MC receptors that have been implicated in MC activation, such as high-affinity IgG receptors or Mas-related G protein-coupled receptor X2 (MRGPRX2) (Fig. 1; Table 1) [92–96]. However, the precise impact of IgG receptors or MRGPRX2 on MC activation in SM contexts remains at present unknown [93–96].

In SM patients with IgE-dependent allergies, several different triggers (allergens) may be detected. The most prevalent allergens producing severe MC activation in SM patients are Hymenoptera venoms [83–85, 97]. However, also other allergens and triggers may provoke anaphylaxis in patients with SM. There are also several mechanisms and molecules that counteract IgE-dependent activation and mediator-release in neoplastic MC. For example, several MC products, such as tryptase and chymase, exert suppressive effects on IgE-dependent MC activation due to their ability to degrade IgE and also allergens to functionally inactive products [98, 99]. The soluble form of the IgE receptor, another MC product, may also be able to counteract IgE-dependent activation and mediator release in MC [100]. However, the clinical impact of these suppressive (activation-limiting) factors and molecules in patients with SM remains at present unknown. Another important aspect is the potential impact of MC mediators on other innate and adaptive immune cells, particularly Th2 and regulatory T lymphocytes which may, respectively, trigger or suppress type II responses, and thereby modulate allergic reactions and anaphylaxis in non-advanced SM patients [101, 102].

In the following paragraphs, the impact of genetic, patient-related and disease-related variables on MC and BA activation in SM is reviewed.

### Genetic Impact: Hereditary Alpha Tryptasemia (HαT)

HαT is a genetic pattern defined by an increased copy number of the *TPSAB1* gene encoding alpha tryptase [103–106]. In the general population, the prevalence of HαT amounts to approximately 5% [104–106]. By contrast, in patients with SM, the prevalence of HαT is higher (roughly 15–17%) [104–106]. This may in part be due to a selection process in which symptomatic SM patients without skin lesions (BMM) accumulate. Indeed, the prevalence of HαT is particularly high among BMM cases (roughly 20–25%) [107]. HαT is also associated with more severe symptoms of MC activation in patients with SM and those with certain allergies [104–106]. The most severe symptoms of MC activation (life-threatening anaphylaxis) are often recorded in patients who have SM as well as an IgE-dependent allergy, with or without HαT [103–105]. However, many individuals with HαT, including some with concomitant SM, are asymptomatic or suffer only from mild symptoms [107, 108]. Correspondingly, the frequency of anaphylactic episodes recorded in SM during follow up has been reported to be identical in SM with and without HαT [107]. Finally, it remains unknown whether and how HαT contributes to MC activation and MC expansion in SM contexts. Regarding tryptase, the total amount per MC may be slightly higher in patients with HαT compared to patients without HαT [105, 106]. However, data published so far suggest that other MC mediators, such as histamine or PGD2, are not produced or stored at higher levels in MC of HαT+ cases compared to healthy controls [103].

### Correlation Between the SM Variant and Severity of MC Activation

In SM, the total amount of mediators in individual patients correlates with the body burden of MC. During an anaphylactic reaction, some or most of these mediators, including histamine, are released from MC and BA, producing clinical signs and symptoms of anaphylaxis [5–9, 43, 50, 52–54]. The severity of the reaction depends on the numbers of MC involved, patient-related factors and comorbidities, the target organ(s) affected, and the trigger (e.g., allergen) that induces mediator release. In general, anaphylactic reactions are more severe in those SM patients who have a very high MC burden and thus higher basal serum tryptase levels [109–111]. However, this is not always the case. In a recent multicenter study, a tryptase level < 90 ng/mL, and BM MC infiltration < 15% were identified as predictors of hypersensitivity reactions, in particular against Hymenoptera venoms, in a larger patient series [112]. Many of these patients may suffer from BMM. Indeed, a remarkable phenomenon is that severe anaphylactic reactions (often against Hymenoptera venoms) are recorded frequently in patients suffering from BMM, a subset of SM characterized by a low burden of neoplastic MC (often with mature MC phenotype) and absence of skin lesions [113–116]. The underlying mechanisms of these surprisingly strong hypersensitivity reactions have not been clarified yet. For example, it is not known whether the high prevalence of HαT in patients with BMM contributes to increased hyperreactivity of MC. It also remains uncertain whether activation of clonal or non-clonal BA and/or or non-clonal MC play a role in the anaphylactic reactions in BMM. Another interesting aspect is that KIT D816V promotes the oncogenic machinery in MC but may even decrease IgE-dependent releasability which has been demonstrated in IgE receptor-bearing ROSA cells, a rather mature human MC line derived from MC-committed cord blood progenitors [117]. These observations have led to the hypothesis that normal (non-neoplastic) MC (carrying wild type KIT) and BA are sometimes involved in the anaphylactic reactions seen in patients with non-advanced SM including BMM. By contrast, in patients with advanced SM, drug hypersensitivity may be a more prevalent cause of anaphylaxis [112].

### Avoiding and Blocking MC Activation in SM: Basic Principles and Role of KIT-targeting Drugs

**Fig. 2:**
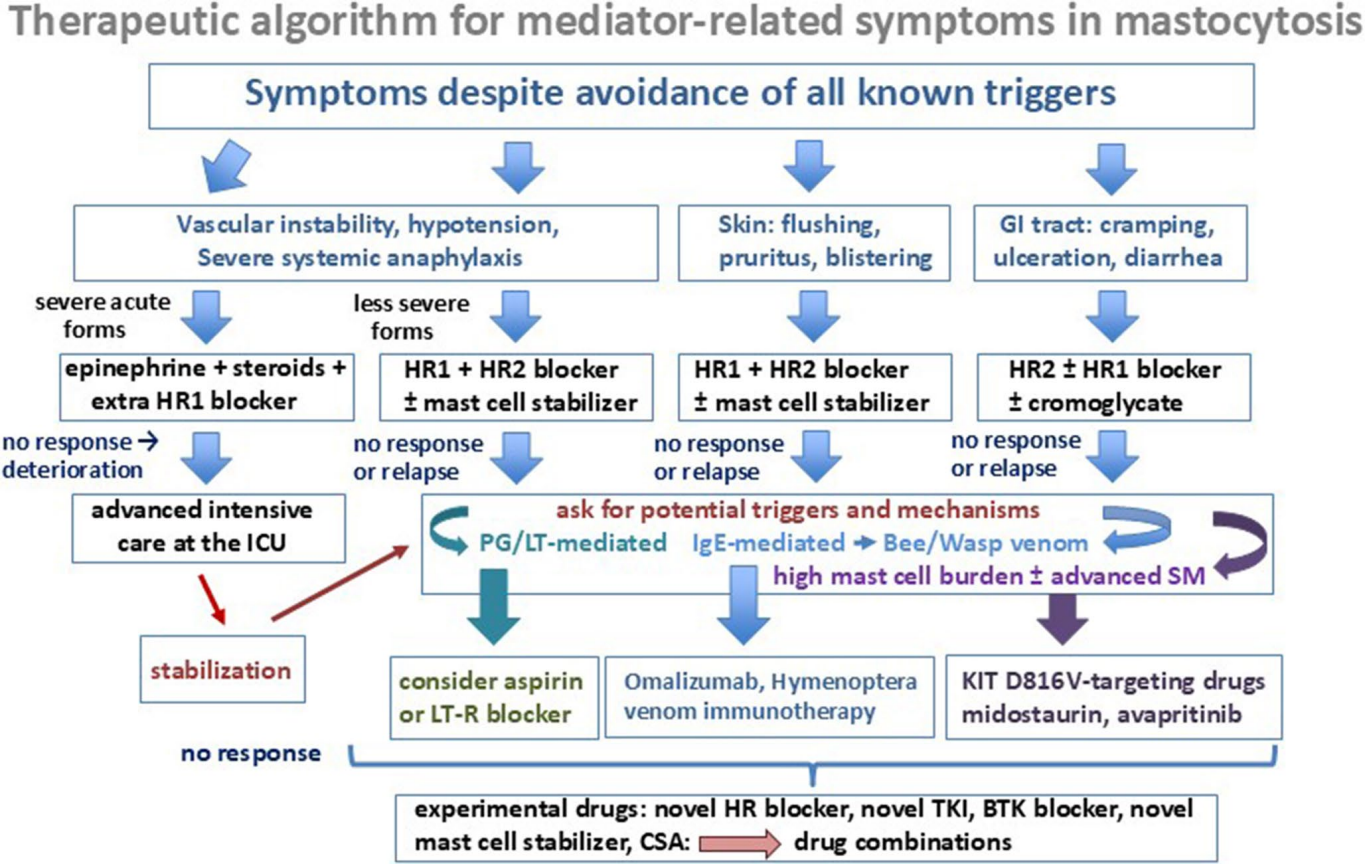
Therapeutic algorithm for patients with mediator-related symptoms in mastocytosis In symptomatic patients with mastocytosis, a stepwise approach is recommended. Before considering specific therapy, patients are adviced to strictly avoid any known or potential trigger of mast cell (MC) activation. If this does not work, specific therapy is recommended. Depending on the severity of the symptoms and the organ-systems involved, the approach starts with first-line treatment aimed at controlling mediator-related symptoms using histamine receptor (HR) type 1 (HR1) and/or type 2 (HR2) antihistamines, with or without (additional) MC stabilizer, such as ketitofen or disodium cromoglycate or leukotriene-targeting drugs. Usually, disodium cromoglycate is only offered to patients with gastrointestinal (GI) symptoms because the drug is not absorbed in the GI tract. In case of severe life-threatening anaphylaxis patients receive glucocorticosteroids and/or epinephrine, including an outpatient prescription for autoinjector therapy. In those who cannot be stabilized, intensive care (IC) at an IC unit (ICU) is required. After stabilization and in all other cases with anaphylaxis, a search for potential triggers and mechanisms should be initiated; and based on identified etiologies, more specific therapy may be offered. In patients with elevated prostaglandin D2 metabolites (measured in uriny samples) acetyl salicylic acid (aspirin) may be recommended. In those with a Hymenoptera venom (usually bee or wasp venom) allergy, specific life-long immunotherapy is usually recommended and in case of resistant anaphylaxis, IgE-targeting therapy with omalizumab has to be considered. In those with a high MC burden or even advanced systemic mastocytosis (SM), MC-eradicating therapy with KIT D816V-targeting drugs (midostaurin, avapritinib) is often recommended. Imatinib may be considered in cases with SM lacking the *KIT* D816V mutation. Sometimes, depending on the overall situation in each case, several of the above mentioned drugs have to be combined, for example HR1 blocker with specific immunotherapy, omalizumab, and/or a KIT D816V-targeting TKI (example: advanced SM with concomitant severe anaphylaxis and a known wasp venom allergy). In the case of resistance against all these drugs, novel experimental drugs should be considered and applied, preferably in the context of a clinical trial. Abbreviations: IgE, immunoglobulin E; PG, prostaglandin; LT, leukotriene; LT-R, LT receptor; CSA, cyclosporin A; BTK, Bruton´s tyrosine kinase

Usually, patients with SM are advised to avoid conditions, substances and environmental factors that could provoke a hypersensitivity reaction [43, 48, 50–54]. Several of these conditions and factors may be patient-specific. In addition, most patients are advised to take continuous prophylactic therapy (preferred first line therapy: histamine receptor blockers) to avoid anaphylaxis (Fig. 2) [43, 48, 50–54]. When considering the development of KIT-related or IgE-related therapeutic strategies aimed at avoiding or blocking MC activation and thus the occurrence of anaphylaxis in patients with SM, the following basic approaches have to be considered (Table 4).

**Table 4:** Pharmacologic strategies to avoid or block mast cell (MC) activation in patients with KIT D816V+ systemic mastocytosis (SM) and severe anaphylaxis

Concept	Available drugs	Therapeutic aim
Targeting MC activation	KIT D816V TKI Corticosteroids Cyclosporin A*	Suppressing mediator release
Targeting MC production and expansion	Midostaurin Avapritinib	Mast cell mass reductionmast cell eradication(complete remission)
Targeting IgE	Omalizumab	IgE reduction/depletion(removing specific IgE)
Targeting mast cell-derived mediators	HR blockers LT/PD synthesis or receptor inhibitors	Suppressing the effectsof mast cell-derivedmediators
* Cyclosporin A is not used in clinical practice in patients with SM. Abbreviations: *TKI* tyrosine kinase inhibitors, *IgE* immunoglobulin E, *LT* leukotriene, *PD* prostaglandin		

First, depletion of normal and neoplastic MC using KIT-targeting drugs is an almost curative maneuver, since MC are obviously more relevant to anaphylaxis than BA in SM contexts. It is also important to know in this regard that the TKI applied currently in SM have sufficient activity against wild type KIT, especially at higher doses, to also suppress the development of normal (non-clonal) MC [60, 65, 72]. However, it is also important to note that imatinib and masitinib can only eliminate normal (non-clonal) MC and MC carrying some mutant forms of KIT, but cannot eliminate KIT D816V+ MC as KIT D816V confers resistance against these TKI [48–50, 60]. Finally, it is important to state that avapritinib can induce complete or near complete hematologic remission in a subset of patients (around 20–30%) with smoldering or advanced SM [67–70]. Therefore, avapritinib and other similar potent new KIT TKI may be the preferred drugs when considering MC depletion as major approach to stop SM-related anaphylaxis in patients. It should also be pointed out that the recommended starting dose of avapritinib used for non-advanced SM is substantially lower than that in advanced SM (25 mg vs. 200 mg daily), and adverse effects due to wild type KIT inhibition are usually not seen at lower doses. Currently, there is not sufficient information to use avapritinib as first line approach in non-advanced SM solely to suppress anaphylaxis, particularly in those with concurrent IgE mediated allergies.

Another approach is to take out specific IgE which may support basic prophylactic therapies, especially when the patient is suffering from an IgE-dependent allergic disease (Fig. 2). The standard approach is to offer treatment with an anti-IgE antibody, like omalizumab [118–120]. A more investigational approach is to block the signaling cascades downstream of KIT or of the IgE receptor using more or less specific drugs. These include the Bruton´s tyrosine kinase (BTK) inhibitor ibrutinib [121], dasatinib, a drug directed against wild type KIT and KIT D816V, BTK and other targets [122], or midostaurin, a multi-kinase inhibitor with a broad range of targets including KIT D816V [65, 71, 72]. Interestingly, midostaurin blocks wild type KIT and KIT D816V as well as several KIT-downstream and IgE receptor-downstream signaling molecules, including SYK [64, 72]. As a result, midostaurin counteracts IgE-mediated (allergen-induced) mediator secretion in normal and neoplastic BA and MC [71, 72]. The effect of midostaurin on IgE-dependent histamine release was also demonstrable in ex vivo BA obtained from SM patients receiving midostaurin [72]. Avapritinib also counteracts IgE-dependent BA activation and histamine release, but does not block SYK or other critical targets in BA or MC [123]. Therefore, the impact of avapritinib on BA and MC activation may be due to the very strong effects of this drug on KIT D816V-dependent signaling [123]. Two observations support this hypothesis. First, it is well known that SCF-induced activation promotes IgE-dependent mediator release in MC [25–27]. Second, avapritinib only blocks IgE-dependent histamine release in BA in patients with SM but not in BA obtained from healthy donors [123]. Whether other novel TKI directed against KIT D816V, such as bezuclastinib or elenestinib, can suppress activation of BA and MC remains unknown.

It also remains unknown whether the suppressive effects of these TKI on BA activation and/or MC activation are both clinically relevant in SM contexts. Based on the overwhelming impact of MC activation in these patients, we believe that it is most important that such drugs can target MC activation in patients. Correspondingly, both TKI applied in clinical practice in SM, namely midostaurin and avapritinib, exert rapid effects on mediator-related symptoms and the quality of life in these patients [64–69].

Finally, a potent approach is to apply conventional anti-allergic drugs directed against mediator production in BA and MC (glucocorticosteroids or cyclosporin A = CSA), mediator secretion in BA and MC (MC stabilizers) or mediator effects, such as histamine receptor (HR) blockers [8, 43, 50, 52–54]. Table 4 shows a summary of drug classes that can be offered to SM patients with severe mediator-related problems and Fig. 2 provides a therapeutic algorithm for these patients.

### Therapeutic Algorithms: Will KIT-targeting TKI Emerge in the Frontline of Treatment of Severe Mediator-induced Symptoms?

Several lines of evidence suggest that KIT TKI exert major beneficial effects in patients with SM who are suffering from the impact of MC mediators produced in the context of MC activation [64–69, 76–80]. Beneficial effects were recorded, both in patients with advanced SM and those with non-advanced SM. In addition, these TKI effects were seen in patients who received these drugs as frontline agents but also in those who were resistant against other anti-mediator type drugs [64–69, 76–80].

Based on these observations, novel TKI directed against KIT D816V are recommended for the treatment of SM patients with severe, drug resistant, mediator-related symptoms and anaphylaxis. However, these TKI can also produce side effects [64–69, 73–80, 124]. Therefore, most experts believe that KIT D816V-targeting drugs should be offered to such patients in second or third line, after resistance or suboptimal response to anti-mediator drugs has been documented, or if first line drugs cannot be tolerated by the patients, whereas first line therapy with such KIT TKI to suppress MC activation is recommended only in special circumstances, for example in those with evidence for an advanced SM or a very high burden of MC such as in SSM [73–77]. However, even in these patients, KIT D816V TKI should be combined with other anti-allergic drugs, including histamine receptor (HR) blockers.

In current treatment algorithms, first line of therapy usually consists of a combination of HR1 and HR2 blockers and strict avoidance of all potential triggers that may provoke MC activation (Fig. 2) [8, 43, 52–54]. In those with Hymenoptera venom allergy, venom immunotherapy is also regarded standard prophylactic therapy (Fig. 2) [83, 84]. MC stabilizers such as disodium cromoglycate or ketotifen and leukotriene receptor blockers, may be considered in those with suboptimal response to H1 and H2 blockers (Fig. 2) [8, 43, 52–54]. In several centers, disodium cromoglycate is also regarded as first line therapy (Fig. 2). In those with a severe IgE-dependent allergy and drug-resistant anaphylaxis, IgE-targeting with omalizumab or other similar agents may be recommended (Fig. 2) [52–54, 118–120].

Second or third-line therapy may also involve a strong KIT D816V inhibitor, such as midostaurin or avapritinib (Fig. 2) [52–54, 124]. It should be noted that midostaurin is not approved for treatment of non-advanced disease, and that gastrointestinal side effects may limit the use of this drug in SM contexts. In rare SM patients without KIT D816V, particularly cases of SM with mutations leading to a spontaneous dimerization of KIT, imatinib may be considered [79–81, 124]. Short-term therapy with a strong KIT D816V-targeting drug may also suppress MC and BA activation and thus mediator release, whereas long-term treatment may result in MC eradication, and may thereby lead to long-term anaphylaxis-free survival and improvement of the quality of life.

## Summary and Future Perspectives

In patients with SM, mediator-related symptoms remain a major clinical challenge, especially when a concomitant IgE-dependent allergy is present. Additional factors, such as co-morbidities, alterations in the immune system, certain infections, or HαT, may also contribute to the severity of hypersensitivity reactions in such cases. When criteria of a MC activation syndrome (MCAS) are fulfilled, the diagnosis of a primary (clonal) MCAS is appropriate. BA may also participate in anaphylactic reactions in SM, but compared to MC, their clinical impact is usually minor. Several different therapeutic strategies have been developed to counteract severe symptoms produced by MC activation products in patients with SM, including prophylactic therapy with mediator-targeting drugs, IgE-targeting drugs, or specific immunotherapy, especially when a Hymenoptera venom allergy is also present. A new emerging concept is to eliminate normal and neoplastic MC in high-risk patients by long-term treatment with novel potent KIT D816V-targeting drugs like midostaurin or avapritinib. In addition, most of these drugs also block MC activation induced by IgE-dependent mechanisms. Whether these drugs can completely eradicate MC and BA, and thereby all symptoms in all patient subsets with symptomatic SM, remains to be elucidated in clinical studies. Other strategies are to combine KIT-targeting TKI with other drugs used to treat patients with MC activation-related symptoms, such as corticosteroids, mediator-targeting drugs or immunotherapy.

## Key References

- Valent P, Akin C, Hartmann K, Nilsson G, Reiter A, Hermine O, et al. Mast cells as a unique hematopoietic lineage and cell system: From Paul Ehrlich’s visions to precision medicine concepts. Theranostics 2020;10:10743-68. 10.7150/thno.46719.
  This article provides a comprehensive overview of biological, phenotypic, and molecular aspects relevant to the human mast cell lineage.
- Valent P, Akin C, Sperr WR, Horny HP, Arock M, Metcalfe DD, Galli SJ. New insights into the pathogenesis of mastocytosis: emerging concepts in diagnosis and therapy. Annu Rev Pathol. 2023;18:361 − 86. 10.1146/annurev-pathmechdis-031521-042618.
  This article provides a contemporary review of molecular, cellular, immunological, and and pathological features in various forms of mastocytosis.
- Akin C. Tyrosine kinase inhibitors in non-advanced systemic mastocytosis. Immunol Allergy Clin North Am. 2023;43(4):743–750. 10.1016/j.iac.2023.05.001. PMID: 37758410.
  This article provides a comprehensive review of the clinical use and perspectives of new KIT D816V-targeting drugs in non-advanced mastocytosis.
- Akin C, Arock M, Carter MC, George TI, Valent P. Mastocytosis. Nat Rev Dis Primers. 2025;11(1):30. 10.1038/s41572-025-00611-8. PMID: 40274818.
  This review article provides a contemporary review of the epidemiology, pathogenesis, diagnosis, classification, prognostication and therapy of mastocytosis.

## Data Availability

No datasets were generated or analysed during the current study.

## Abbreviations

ASM: Aggressive SM
BA: Basophils
BM: Bone marrow
BMM: Bone marrow mastocytosis
BTK: Bruton´s tyrosine kinase
C5aR: Complement receptor 5a
CM: Cutaneous mastocytosis
GI: Gastrointestinal
HαT: Hereditary alpha tryptasemia
HR: Histamine receptor
IgE: Immunoglobulin E
FceRI: Fc-epsilon receptor I
IL: Interleukin
ISM: Indolent systemic mastocytosis
LT: Leukotriene
MC: Mast cell
MCAS: Mast cell activation syndrome
MCL: Mast cell leukemia
MRGPRX2: Mas-related G protein-coupled receptor X2
PD: Prostaglandin D2
SCF: Stem cell factor
SM: Systemic mastocytosis
SM-AHN: SM with associated hematologic neoplasm
SSM: Smoldering SM
SYK: Spleen tyrosine Kinase
TKI: Tyrosine kinase inhibitors
TNF: Tumor necrosis factor
VEGF: Vascular endothelial growth factor
WT: Wild type
WDSM: Well differentiated SM
WHO: World Health Organization
